# Evaluating the Impact of Near-Natural Restoration Strategies on the Ecological Restoration of Landslide-Affected Areas Across Different Time Periods

**DOI:** 10.3390/plants14152331

**Published:** 2025-07-28

**Authors:** Sibo Chen, Jinguo Hua, Wanting Liu, Siyu Yang, Wenli Ji

**Affiliations:** 1 College of Landscape Architecture and Arts, Northwest A&F University, Xianyang 712100, China; chensibo@nwafu.edu.cn (S.C.);; 2 College of the Environment and Ecology, Xiamen University, Xiamen 361102, China

**Keywords:** landslide, restoration strategies, ecological succession, vegetation characteristics, soil properties

## Abstract

Landslides are a common geological hazard in mountainous areas, causing significant damage to ecosystems and production activities. Near-natural ecological restoration is considered an effective strategy for post-landslide recovery. To investigate the impact of near-natural restoration strategies on the recovery of plant communities and soil in landslide-affected areas, we selected landslide plots in Lantian County at 1, 6, and 11 years post-landslide as study sites, surveyed plots undergoing near-natural restoration and adjacent undisturbed control plots (CK), and collected and analyzed data on plant communities and soil properties. The results indicate that vegetation succession followed a path from “human intervention to natural competition”: species richness peaked at 1 year post-landslide (D_m_ = 4.2). By 11 years, dominant species prevailed, with tree species decreasing to 4.1 ± 0.3, while herbaceous diversity increased by 200% (from 4 to 12 species). Soil recovery showed significant temporal effects: total nitrogen (TN) and dehydrogenase activity (DHA) exhibited the greatest increases after 1 year post-landslide (132% and 232%, respectively), and by 11 years, the available nitrogen (AN) in restored plots recovered to 98% of the CK levels. Correlations between plant and soil characteristics strengthened over time: at 1 year, only 6–9 pairs showed significant correlations (*p* < 0.05), increasing to 21–23 pairs at 11 years. Near-natural restoration drives system recovery through the “selection of native species via competition and activation of microbial functional groups”. The 6–11-year period post-landslide is a critical window for structural optimization, and we recommend phased dynamic regulation to balance biodiversity and ecological functions.

## 1. Introduction

Landslides are a natural geomorphic process that severely disrupts mountain ecosystems [1,2,3]. Their occurrence and causes involve multiple factors, including topography, soil structure, groundwater levels, geological structures, climate, and human activities. Landslides reshape the terrain and alter mountain topography, leading to the burial or destruction of existing vegetation [4,5]. They accelerate soil erosion, alter the physical and chemical properties of the soil, and reduce its fertility and water retention capacity [6,7]. Additionally, landslides destroy local biodiversity, thereby impacting plant growth and ecosystem productivity. They may also destabilize ecosystems, making them more susceptible to other natural disasters, and pose significant threats to the safety and property of local residents [8].

Landslide ecological succession is an integral part of forest succession, playing a crucial role in maintaining species diversity by disrupting the ecological dominance of certain species [9,10]. Landslides occurring in different regions and under different restoration approaches display varied vegetation succession trajectories [11,12]. For instance, landslides in the Mgeta Valley of the Ulugulu Mountains in Tanzania demonstrate substantial vegetation recovery potential, with plant communities on the landslide surface becoming remarkably similar to those on adjacent grazing lands after approximately 83 weeks [1]. Conversely, landslides on the Ninole Ridge in Hawaii, due to the loss of tephra-derived soils, have triggered invasions by exotic species, which subsequently create favorable conditions for further invasions by other exotic species [13]. The succession trajectories of plant communities in landslide-disturbed areas are significantly influenced by different post-disaster recovery methods and patterns of human disturbance [14]. Therefore, selecting an appropriate ecological restoration philosophy following a landslide is a critical consideration.

The current research on post-landslide restoration primarily focuses on two approaches: first, natural recovery pathways, as demonstrated by Neto et al. (2017), which indicate that ecosystems possess self-repair capabilities but require decades for recovery [15]; second, engineered restoration, as studied by Kang et al. (2022) in the Longxi-Hongkou National Nature Reserve in China, where engineered restoration rapidly stabilizes slopes but leads to a decline in biodiversity [16]. The concept of near-natural restoration has long been a focus in the field of ecological recovery [17,18]. As early as the 19th century, Germany utilized different species and age classes of suitable tree species to restore mixed forests that closely resemble natural forests, establishing a “near-natural forestry” management model through near-natural ecological restoration [19]. Japanese ecologist Akira Miyawaki developed the “Miyawaki method” of “building native forests with native tree species”, which has been widely applied and promoted across multiple countries [20]. Near-natural ecological restoration is a method that “learns from nature”, emphasizing that the restored ecosystem should exhibit similarities and integration with surrounding natural ecosystems, distinguishing it from traditional artificial restoration approaches [17,21,22].

Near-natural restoration following landslides typically employs techniques such as substrate improvement, selection of excellent native pioneer plants, optimization of plant functional configurations, and utilization of soil seed banks, with the goal of achieving self-sustaining operation in landslide-disturbed areas [23,24]. Tailored to local conditions, this approach implements targeted measures and precise efficiency to effectively restore ecosystem stability and diversity [25]. In post-disaster ecosystems, near-natural restoration not only enhances productivity and improves ecosystem functions and services but also accelerates the ecosystem succession cycle during recovery [26,27,28]. While near-natural restoration is regarded as an effective strategy for post-landslide recovery, the long-term effects of adopting such strategies on the recovery of plant communities and soil quality in landslide-disturbed areas remain uncertain.

This study focuses on landslide-disturbed areas in Lantian County, investigating near-natural restoration plots affected by landslides in 2011, 2016, and 2021. Continuous observations and surveys were conducted in 2022 and 2023, with adjacent undisturbed plots selected as controls. Compared to other studies, this research is distinguished by two key aspects: first, it overcomes the limitation of single-time-point analyses by examining the changes in near-natural restoration effects from 1 to 11 years post-landslide; second, it elucidates the vegetation–soil feedback mechanisms during the near-natural recovery process following landslides. This research examines the ecological recovery effects of near-natural restoration in landslide-affected areas with the following primary objectives: (1) To investigate changes in plant communities and diversity in landslide plots under near-natural restoration across different time gradients. (2) To assess changes in soil properties and their correlations with plant recovery as restoration time progresses. (3) To explore whether near-natural restoration strategies can establish a relatively stable ecosystem in landslide-affected areas. The findings of this study offer references and recommendations for ecological restoration following landslides, contributing to the refinement and optimization of post-landslide ecological restoration methods. By providing multifaceted guidance for post-landslide environmental restoration, this work holds significant practical importance for the protection and recovery of ecosystems in disaster-affected regions.

## 2. Materials and Methods

### 2.1. Study Area

**Figure 1:**
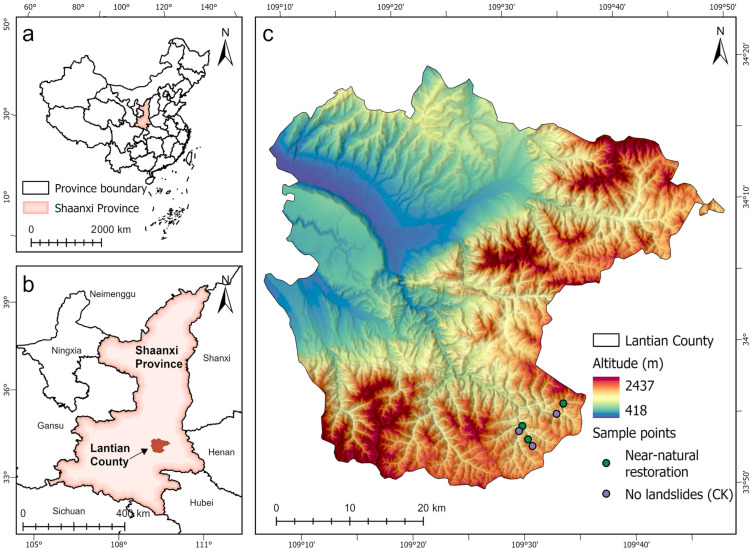
Research scope map. (**a**) The location of Shaanxi Province in China. (**b**) Lantian County’s location in Shaanxi Province. (**c**) The study area and unaffected study area’s location in Lantian County.

Lantian County (33°53′44″ N–34°4′56″ N, 109°33′57″ E–109°16′48″ E) is located in the southeastern part of Xi’an City, Shaanxi Province, China, approximately 45 km from Xi’an. It is bordered by the Hengling Mountains to the north and the Qinling Mountains to the south. The region experiences a warm temperate continental semi-humid climate with four distinct seasons and synchronous rainfall and heat periods. Its average annual temperature ranges from 12.0 to 13.6 °C, with an average annual sunshine duration of 2148.8 h and a frost-free period exceeding 212 days. Precipitation in Lantian County is primarily concentrated between July and October, with an annual average of 726 mm. Rainfall distribution is uneven throughout the year and exhibits interannual variability, with spatial differences characterized by higher precipitation in the eastern part and lower in the western part [29,30]. The county’s soil types are diverse, encompassing 107 soil species and 10 soil classes, including gray soil, brown soil, red soil, and yellow soil. The primary bedrock consists of gravel and clay, though soil composition and nutrient content may vary locally. Lantian County falls within the warm temperate deciduous broadleaf forest zone, with the dominant vegetation type being mixed coniferous–broadleaf forests, covering 44.7% of the area. Situated on the northern slopes of the Qinling Mountains, mountainous terrain constitutes 56.1% of the county’s total area, while forests and wastelands account for 62.8% of the land (as shown in Figure 1) [31].

### 2.2. Sample Plot Overview

This study selected landslide-affected areas in Lantian County from 2011, 2016, and 2021 for investigation, with a 5-year interval between each time gradient. Post-landslide ecological recovery typically includes a rapid recovery phase (1–5 years), a stabilization phase (5–10 years), and a late successional phase (>10 years), with the 5-year interval effectively capturing key transitions across these stages. For instance, the 2021 plots may still be in the rapid recovery phase, while the 2011 plots may show early signs of advanced communities. Surveys were conducted in October 2022 and October 2023, as this period corresponds to the peak of annual plant growth. The study area included three near-natural restoration plots (each surveyed once in 2022 and once in 2023) and three nearby control plots unaffected by landslides (also surveyed once in 2022 and once in 2023). This design adheres to the core principles of ecological control experiments. Adjacent control plots provide an undisturbed baseline under local environmental conditions, serving to (1) quantify initial ecological losses due to landslides (e.g., biomass reduction rates); (2) define recovery target values (e.g., species diversity recovery levels); and (3) eliminate confounding effects of regional environmental variables (e.g., climate, topography, soil baseline).

The 2021 landslide plot surveyed in October 2022 was designated as 1N, and the same plot surveyed in October 2023 was designated as 1NN, with the other plots following the same naming convention (see Table 1 for details). The initial survey, conducted in October 2022, covered six plots—three near-natural restoration plots and three adjacent control plots—with a total of 126 sampling points. A follow-up survey was conducted one year later in October 2023, also covering 126 sampling points across the same six plots. This study comprised a total of 12 plot surveys (plot details in Table 1, each plot with an area exceeding 1000 m^2^) and 252 sampling points. The data collected included plant functional traits, plant community composition, environmental factors, and soil conditions. The Lantian County Natural Resources Bureau provided specific restoration years and coordinates for the landslide plots, which were cross-verified through communication with local villagers. Each landslide plot was divided into three sections—the upper slope, middle slope, and lower slope—to investigate differences in vegetation recovery across slope positions, with each plot’s area exceeding 1000 m^2^ (see Table 1 for plot details).

### 2.3. Data Collection

**Figure 2:**
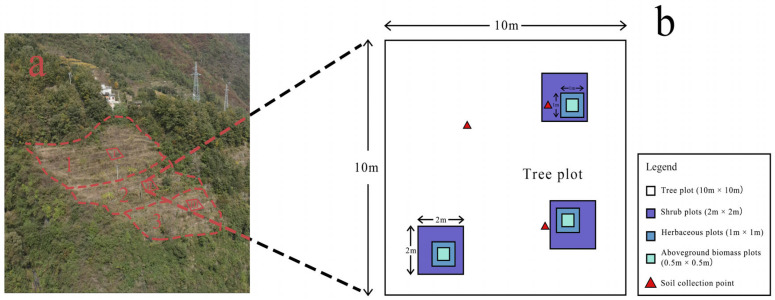
Sampled plot arrangement plan. (**a**) Landslide site diagram. Layout of the plots: (1) upper slope; (2) middle slope; (3) lower slope. In (**b**), I, II, and III represent three 10 m × 10 m tree plots, while the sizes of the other plots can be referred to in the legend.

Based on the internationally recognized Braun-Blanquet stratified sampling protocol, we used plots of varying sizes to sample vegetation data: tree plots measured 10 m × 10 m (three tree plots randomly established per slope position, one each for upper, middle, and lower slopes); shrub plots measured 2 m × 2 m (nine shrub plots per slope position, three each for upper, middle, and lower slopes); and herbaceous plots measured 1 m × 1 m (nine herbaceous plots per slope position, three each for upper, middle, and lower slopes) (see Figure 2 for plot layout details). The minimum distance between the plots was 10 m. Plant community composition was documented by identifying and recording all vascular plant species within each plot. Plant functional traits were measured as follows: for trees, height (H, m; tape measure/laser rangefinder), diameter at breast height (DBH, cm; calipers), and canopy closure (CD, %; Braun-Blanquet scale); for shrubs, height (H, cm), basal diameter (DBH, mm; calipers), and coverage (COV, %; Braun-Blanquet scale); and for herbaceous species, height (H, cm) and abundance (NO.). Additionally, 3–5 mature leaves per species were collected across all plots for laboratory determination of leaf functional traits, leaf area (LA), leaf thickness (LT), and dry weight (DW), to calculate derived traits like specific leaf area (SLA). All aboveground herbaceous biomass within the 1 m × 1 m plots was harvested; wet weight (WW) was recorded in the field and dry weight (DW) after oven-drying to determine aboveground biomass (W, g/m^2^) [30,32].

Environmental factors inherent to the sampling design included slope position (upper, middle, lower). Soil conditions were assessed by collecting minimally disturbed soil cores (0–30 cm depth, 5 cm diameter) with care to preserve aboveground vegetation. Three composite soil samples per slope position were created by mixing soil from different depths within that position. Each composite sample was split: one portion for immediate field measurement of soil moisture content (SMC; drying method), the other sealed for laboratory analysis. Laboratory analyses determined the following: soil chemical properties—total nitrogen (TN; Kjeldahl method), total phosphorus (TP; antimony anti-colorimetric method), total potassium (TK; sodium carbonate fusion method), available phosphorus (AP; antimony anti-colorimetric method), available potassium (AK; NH4OAc extraction flame photometry), alkali-hydrolyzable nitrogen (AN; alkali diffusion method), and cation exchange capacity (CEC; NaOAc method); and soil enzyme activities—dehydrogenase (DHA; TTC colorimetric method), protease (PRO; Roberts copper method), urease (UE; indophenol blue method), phosphatase (PHO; phenyl phosphate colorimetric method), and sucrase (SUC; 3,5-dinitrosalicylic acid method) [33,34,35].

### 2.4. Data Analysis

The Shapiro–Wilk test was applied to evaluate the normality of the soil physicochemical properties and vegetation traits across the different restored ecosystems. Where necessary, the data were transformed using logarithmic, square root, or arcsine square root functions to improve normality and ensure the homogeneity of variance. To characterize the structure and diversity of plant communities, several commonly used ecological indices were calculated based on field survey data. These included the Species Importance Value Index (IV), which reflects species dominance through relative abundance, height, and coverage; the Species Richness Index (Dm), which captures the number of species present; the Simpson Dominance Index, which measures species dominance; the Pielou Evenness Index, which evaluates the uniformity of individual distribution among species; and the Shannon–Wiener Diversity Index, which integrates both species richness and evenness to quantify overall diversity [36,37,38]. The detailed calculation methods for these indices are presented in Table 2.

To compare soil and vegetation characteristics among different restoration stages, independent sample *t*-tests were performed. Pearson correlation coefficients were used to assess linear relationships between soil properties and vegetation traits. All data processing, statistical analyses, and visualizations were conducted using R software (version 4.5.0; R Core Team, 2024, https://www.r-project.org/ (accessed on 15 March 2025)).

## 3. Results

### 3.1. Plant Community Species Composition

The species composition and importance values of the plant communities in the landslide plots under near-natural restoration strategies were analyzed. For each vegetation layer, only the top five species by importance value were calculated (fewer than five species indicate that only those species were present in the layer), as shown in Table 3, Table 4 and Table 5. By comparing data from 2022 and 2023, it is evident that the species composition in the plant communities became more diverse with increasing restoration time, accompanied by corresponding changes in their species importance values.

In the 2021 landslide plots, the dominant species in the tree layer remained consistent between 2022 and 2023 (i.e., plots 1N and 1NN; Table 3, Tree Layer), but their importance values changed. The importance values of *Toona sinensis* and *Zanthoxylum bungeanum* increased (from 15.07% to 23.74% and from 21.14% to 23.01%, respectively), while that of *Pinus armandii* decreased (from 22.42% to 19.96%). The shrub layer had few species (Table 3, Shrub Layer), with minimal changes, adding only *Cornus alba* after one year. In the herbaceous layer (Table 3, Herbaceous Layer), the importance value of *Artemisia annua* decreased (from 30.54% to 23.20%), while *Achnatherum chinense* became the dominant species.

In the 2016 landslide plots, the dominant species in the tree layer remained consistent between 2022 and 2023 (i.e., plots 2N and 2NN; Table 4, Tree Layer). The dominance of *Pinus massoniana* decreased (importance value from 39.26% to 33.59%), while that of *Cotinus coggygria* increased (importance value from 14.38% to 20.39%). The shrub layer gained two additional species, *Lespedeza bicolor* and *Berberis feddeana* (Table 4, Shrub Layer). In the herbaceous layer, *Artemisia lavandulifolia* emerged as the dominant species (Table 4, Herbaceous Layer).

In the 2011 landslide plots, the dominant species in the tree, shrub, and herbaceous layers remained unchanged between 2022 and 2023 (i.e., plots 3N and 3NN; Table 5). In the tree layer (Table 5, Tree Layer), the dominance of *Pinus bungeana*, *Robinia pseudoacacia*, and *Juglans regia* decreased, while that of *Gleditsia sinensis* and *Prunus davidiana* increased. In the shrub layer (Table 5, Shrub Layer), the dominance of *Spiraea × vanhouttei* increased (importance value from 53.82% to 57.95%). In the herbaceous layer (Table 5, Herbaceous Layer), the importance values of *Carex breviculmis*, *Chrysanthemum lavandulifolium*, and *Artemisia argyi* showed slight declines.

### 3.2. Alpha Diversity of Plant Communities

To assess the impact of different restoration periods on the recovery of plant community species diversity, the α diversity indices of plant communities were compared across near-natural restoration plots affected by landslides in 2021, 2016, and 2011, as well as undisturbed control plots (CK). The α diversity indices included the Margalef Richness Index, Simpson Dominance Index, Shannon–Wiener Diversity Index, and Pielou Evenness Index. These indices reflect species richness, dominance, diversity, and evenness of distribution within the communities.

**Figure 3:**
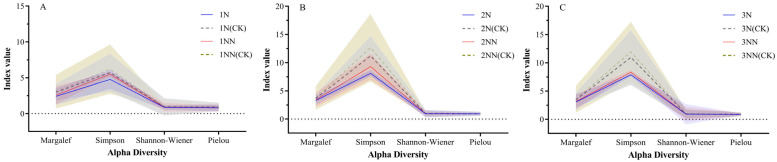
Alpha diversity of plant community species. (**A**–**C**) represent plots affected by landslide events in 2021, 2016, and 2011, respectively. Plots surveyed in 2022 are abbreviated as N, and those surveyed in 2023 as NN. N (CK) and NN (CK) denote respective control plots.

Figure 3 shows the dynamics of α diversity across the restoration stages. The Margalef Richness Index for plots 1NN, 2NN, and 3NN (surveyed in 2023) remained similar to that of plots 1N, 2N, and 3N (surveyed in 2022), indicating stable species richness (Figure 3A–C). The Simpson Dominance Index increased over time, with the largest increase observed in plot 2NN (Figure 3B), suggesting enhanced species dominance. The Shannon–Wiener Diversity Index and Pielou Evenness Index showed minor fluctuations (Figure 3A–C), indicating relatively stable diversity structure. From 1N to 2NN (Figure 3A,B), α diversity increased rapidly, while the increase from 2NN to 3NN (Figure 3B,C) slowed, reflecting an early-stage surge in diversity followed by gradual stabilization under near-natural restoration.

### 3.3. Changes in Plant Community Functional Traits

**Figure 4:**
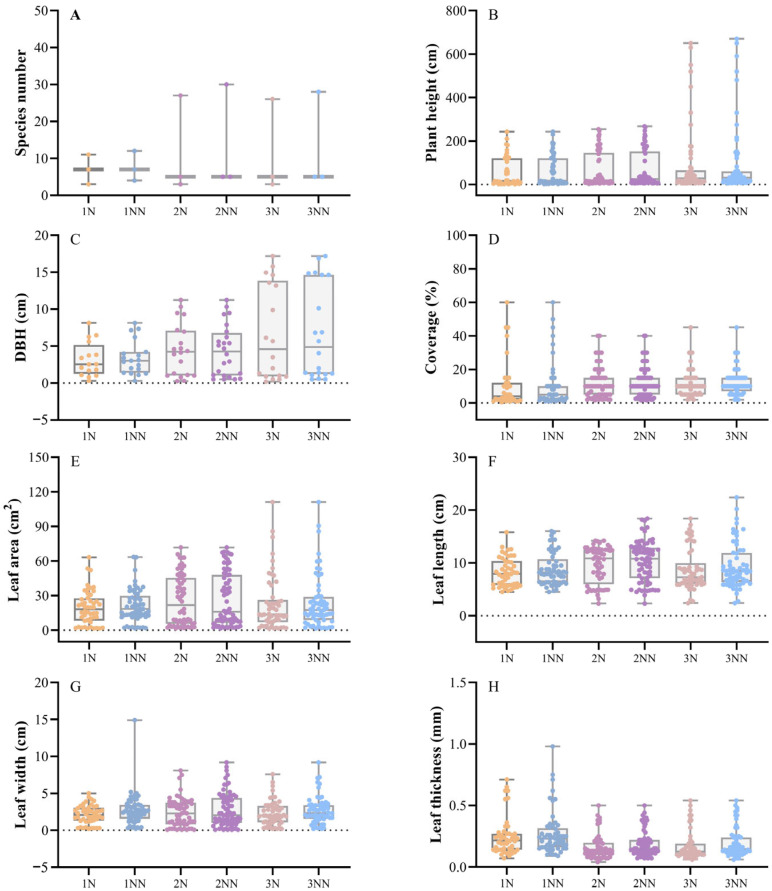
Changes in functional traits of plant communities. Plots 1, 2, and 3 represent areas affected by landslide events in 2021, 2016, and 2011, respectively. N denotes plots sampled in 2022, NN denotes plots sampled in 2023, and CK represents control plots, indicating undisturbed areas without landslides. (**A**) is the number of species in the community; (**B**) is the height of the plant; (**C**) is the diameter at breast height of the plant; (**D**) is the density of the plant; (**E**) is the leaf area; (**F**) is the leaf length; (**G**) is the leaf width; and (**H**) is the leaf thickness.

To investigate the effects of restoration time on the composition, structure, and functional traits of plant communities after landslides, changes in community-level functional traits were examined. The results are shown in Figure 4. The functional trait values in plots 1NN, 2NN, and 3NN (surveyed in 2023) showed slight increases compared to plots 1N, 2N, and 3N (surveyed in 2022).

Species richness peaked in plots 1N and 1NN (Figure 4A), likely due to the planting of multiple native tree species in the early post-landslide stage. With increasing restoration time, dominant species gradually monopolized environmental resources, resulting in reduced species diversity. Similar trends were observed in plant height and DBH (Figure 4B,C), reflecting stronger competitive ability and enhanced growth of dominant species. Near-natural restoration in the short-term post-landslide stage produced higher species diversity (Figure 4A), broader vertical structure, and higher canopy coverage (Figure 4D). Leaf functional traits varied across time gradients. Leaf area and leaf length followed a U-shaped trend (Figure 4E,F). Leaf width remained relatively stable (Figure 4G), while leaf thickness decreased gradually (Figure 4H).

### 3.4. Changes in Soil Properties of Landslide Plots

**Figure 5:**
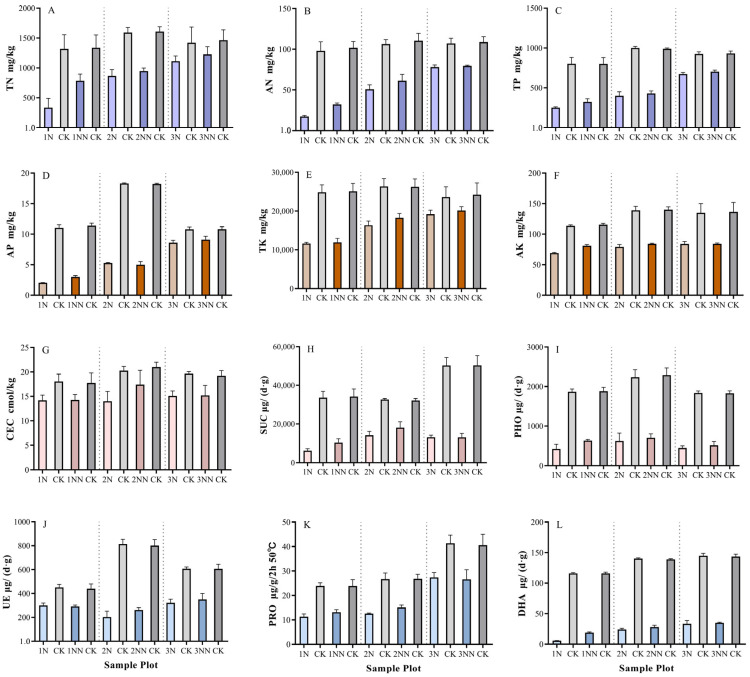
Changes in soil properties in landslide sample sites. Plots 1, 2, and 3 represent areas affected by landslide events in 2021, 2016, and 2011, respectively. N denotes plots sampled in 2022, NN denotes plots sampled in 2023, and CK represents control plots, indicating undisturbed areas without landslides. (**A**) is the total nitrogen content; (**B**) is the available nitrogen content; (**C**) is the total phosphorus content; (**D**) is the effective phosphorus content; (**E**) is the total potassium content; (**F**) is the effective potassium content; (**G**) is the cation exchange capacity; (**H**) is the sucrase content; (**I**) is the phosphatase content; (**J**) is the urease content; (**K**) is the protease content; and (**L**) is the dehydrogenase content.

Soil samples from landslide plots were collected, and the measured soil properties included total nitrogen (TN), available nitrogen (AN), total phosphorus (TP), available phosphorus (AP), total potassium (TK), available potassium (AK), cation exchange capacity (CEC), sucrase activity (SUC), phosphatase activity (PHO), urease activity (UE), protease activity (PRO), and dehydrogenase activity (DHA). The results are presented in Figure 5. The soil property metrics in plots 1NN, 2NN, and 3NN (surveyed in 2023) showed increases compared to plots 1N, 2N, and 3N (surveyed in 2022), with TN, AN, SUC, PHO, and DHA exhibiting the most significant changes (Figure 5A,B,H,I,L).

The mean TN values increased from 340 mg/kg in 1N to 790 mg/kg in 1NN, from 870 mg/kg in 2N to 950 mg/kg in 2NN, and from 1110 mg/kg in 3N to 1230 mg/kg in 3NN (Figure 5A). The mean AN value increased from 17.47 mg/kg in 1N to 32.35 mg/kg in 1NN, from 50.83 mg/kg in 2N to 61.41 mg/kg in 2NN, and from 78.18 mg/kg in 3N to 79.62 mg/kg in 3NN (Figure 5B). The mean SUC values increased from 6220 μg/ (d·g) in 1N to 10,260 μg/ (d·g) in 1NN and from 14,130 μg/ (d·g) in 2N to 18,050 μg/ (d·g) in 2NN, but decreased slightly from 13,130 μg/ (d·g) in 3N to 13,060 μg/ (d·g) in 3NN (Figure 5H). The mean PHO values increased from 430 μg/ (d·g) in 1N to 630 μg/ (d·g) in 1NN, from 620 μg/ (d·g) in 2N to 700 μg/ (d·g) in 2NN, and from 450 μg/ (d·g) in 3N to 510 μg/ (d·g) in 3NN (Figure 5I). The mean DHA values increased from 5.73 μg/ (d·g) in 1N to 19.05 μg/ (d·g) in 1NN, from 24.04 μg/ (d·g) in 2N to 28.00 μg/ (d·g) in 2NN, and from 33.49 μg/ (d·g) in 3N to 35.06 μg/ (d·g) in 3NN (Figure 5L). These results indicate that over longer time gradients (e.g., comparing the 2021 and 2011 landslide plots), soil properties exhibited stronger responses to near-natural restoration, approaching levels observed in the undisturbed control plots (CK). Moreover, during the initial stages of restoration, soil property metrics showed the most rapid increases.

### 3.5. Correlation Between Plant Communities and Soil Physicochemical Properties in Landslide Plots

**Figure 6:**
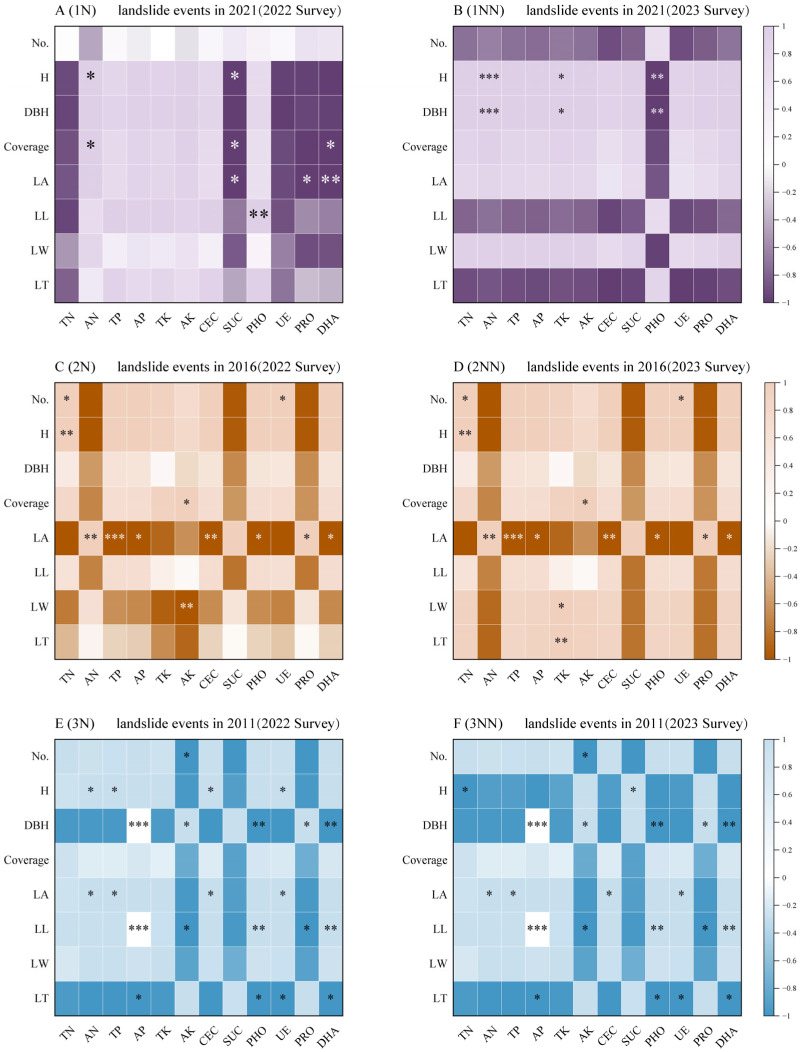
Correlations between plant community characteristics and soil physicochemical properties in landslide plots. Plots 1, 2, and 3 represent areas affected by landslide events in 2021, 2016, and 2011, respectively. N denotes plots sampled in 2022, and NN denotes plots sampled in 2023. Plant traits include the following: No. (species number), H (plant height), DBH (diameter at breast height or basal diameter), coverage (canopy closure or layer coverage), LA (leaf area), LL (leaf length), LW (leaf width), and LT (leaf thickness). Soil physicochemical properties include the following: TN (total nitrogen content), AN (available nitrogen content), TP (total phosphorus content), AP (available phosphorus content), TK (total potassium content), AK (available potassium content), CEC (cation exchange capacity), SUC (sucrase activity), PHO (phosphatase activity), UE (urease activity), PRO (protease activity), and DHA (dehydrogenase activity). Significance levels: * *p* ≤ 0.05; ** *p* ≤ 0.01; *** *p* ≤ 0.001.

Correlation analyses were conducted between plant community functional traits, environmental factors, and soil properties to explore potential relationships in the landslide-affected plots. The results are shown in Figure 6. In the one-year restoration plots, 9 out of 96 data pairs showed significant correlations in 2022 (Figure 6A, *p* < 0.05), decreasing to 6 pairs in 2023 (Figure 6B, *p* < 0.05), indicating weakened relationships over time. In the six-year plots, 12 significant correlations were observed in 2022 (Figure 6C), increasing slightly to 13 in 2023 (Figure 6D). Total potassium (TK) showed stronger correlations with leaf width and thickness over the one-year interval; available potassium (AK) also exhibited an enhanced correlation with leaf width.

In the eleven-year plots, 23 significant correlations were detected in 2022 (Figure 6E), with 21 remaining in 2023 (Figure 6F). Several environmental variables—available nitrogen (AN), total phosphorus (TP), available phosphorus (AP), total potassium (TK), cation exchange capacity (CEC), phosphatase activity (PHO), urease activity (UE), protease activity (PRO), and dehydrogenase activity (DHA)—were consistently correlated with multiple plant functional traits.

## 4. Discussion

### 4.1. Changes in Plant Communities Across Different Time Gradients in Landslide Plots

Changes in plant functional traits serve as key indicators for assessing community stability [39]. This study, through field surveys of landslide-affected areas, elucidates the dynamic succession patterns of plant communities under near-natural restoration. In the short term (1–2 years post-landslide), reliance on techniques such as rapid artificial planting leads to the formation of structurally diverse plant communities in the tree and shrub layers. The herbaceous layer, dependent on soil seed banks and natural dispersal, exhibits low species richness, dominated by pioneer species such as *Artemisia annua* and *Artemisia lavandulifolia* (Table 3). At this stage, species richness (Dm) and diversity (H) are high (Figure 3A), but community stability remains low. In the mid-term (6–7 years post-landslide), dominant tree species (e.g., *Cotinus coggygria*, *Quercus variabilis*) progressively monopolize resources through competition (Figure 4B,C), resulting in a reduction in species richness (Figure 4A). The shrub layer experiences a slight decline in species number due to shading and competition from the tree layer (e.g., shrub species in plot 2NN decreased to four; Table 4). In contrast, the herbaceous layer benefits from improved microhabitats, leading to increased species richness (Table 4), reflecting a transition from rapid human intervention to a natural competitive equilibrium. Over the long term (11–12 years post-landslide), the tree layer evolves toward a few stable dominant species (e.g., *Robinia pseudoacacia*, *Pinus armandii*), with increased plant height and diameter (Figure 5B,C). The shrub layer continues to decline in species richness (Table 5), driven by interspecific competition and resource capture by the tree canopy. Nevertheless, the overall system trends toward greater complexity and stability.

Near-natural restoration, through a dual pathway of “human intervention + natural recovery”, accelerates the transition of plant communities from a pioneer stage to a competitive equilibrium [4]. Previous studies have shown that artificial planting significantly promotes the rapid establishment of plant communities; for instance, in sandy land restoration, planted semi-shrub coverage can reach 17.04% to 22.62%. Furthermore, near-natural restoration typically begins with native or pioneer species, which improve soil conditions and facilitate the establishment of subsequent species. This study found that functional traits such as plant height, diameter at breast height, and leaf area increased with restoration time (Figure 4). These shifts highlight both increased plant adaptability and community reorganization, driven by biotic and abiotic filters. In post-landslide settings, native species with crucial functional traits—such as resource acquisition plasticity, robust root systems for soil stabilization, and tolerance to nutrient limitation—are essential for initiating ecosystem recovery [40,41]. Such traits facilitate rapid establishment and the recruitment of other species by modifying microhabitats through processes like canopy shading, litter deposition, and rhizosphere microbial stimulation. The significant rise in species richness and diversity observed in early-stage near-natural restoration plots likely results from expanded niche availability and reduced anthropogenic pressure, fostering species coexistence via niche complementarity and facilitation [42,43]. However, as communities develop, intensified competition for limited resources leads to competitive exclusion by dominant species, slowing biodiversity accumulation. This trajectory aligns with classical succession theory, where communities later shift towards competition and resource filtering.

### 4.2. Changes in Soil Properties and Their Relationships with Plants Across Different Time Gradients in Landslide Plots

Soil properties are a critical factor influencing plant community recovery, and restoration time significantly affects ecosystem soil characteristics. In this study, the temporal dynamics of soil recovery were evident, with soil nutrients (total nitrogen [TN], available nitrogen [AN], available phosphorus [AP]) and enzyme activities (sucrase [SUC], phosphatase [PHO], dehydrogenase [DHA]) showing significant increases over restoration time (Figure 5). The most substantial increases occurred within 1–6 years post-landslide (e.g., TN increased by 132% in plot 1NN), demonstrating that vegetation recovery drives soil organic matter accumulation and enhances microbial activity. Soil recovery lags behind vegetation recovery, but over longer restoration periods, their synergy strengthens, with enzyme activities (SUC, DHA) serving as key biomarkers of system recovery. Previous studies have shown that unlike the changes in soil physical properties under different time gradients of afforestation, soil structure improves over time, with increased total porosity and enhanced coordination of water, air, and heat, which promote water retention and soil conservation [44,45,46]. Additionally, during natural vegetation recovery, soil physicochemical properties such as bulk density and capillary porosity change over time, indicating the role of restoration duration in improving soil physical conditions [47,48]. However, differences in soil properties across plots with varying restoration periods suggest that soil recovery is a relatively slow process compared to plant recovery, requiring long-term investment and management [49,50]. Concurrently, the degree of soil property improvement directly influences the effectiveness of plant community recovery [51].

Plants and soils form an interdependent, unified system that collectively determines the ecological restoration of landslide-affected areas [52]. Plant roots and soil biota interact physically with the soil, helping to protect it from erosion and stabilize its structure [53,54]. In this study, plant–soil interactions showed weak and highly variable correlations in the short term (Figure 6A,B), reflecting unstable community structures and the absence of effective soil feedback mechanisms. In the mid-term, correlations between potassium (total potassium [TK], available potassium [AK]) and leaf traits (leaf width, leaf thickness) strengthened (Figure 6C,D), indicating that soil nutrient cycling begins to support the expression of plant functional traits. Long-term results revealed significant correlations between nitrogen and phosphorus cycling (AN, total phosphorus [TP], AP), enzyme activities (PHO, DHA), and most plant traits (Figure 6E,F), confirming the establishment of mature plant–soil positive feedback mechanisms and the development of ecosystem self-sustainability. Furthermore, in the absence of precise landslide timing data, plant characteristics and soil properties within landslide-affected areas can be used to infer the time since the landslide event, enabling a better understanding of site conditions and informing more rational restoration decisions [55].

### 4.3. Practical Implications of Optimizing Near-Natural Restoration in Landslide Plots

Near-natural restoration should balance the “timeliness of human intervention” with the “principles of natural succession”, employing phased dynamic management to achieve synergistic recovery of ecological functions and biodiversity [56]. First, prior to restoration, a thorough survey of dominant, fast-growing, and locally adapted species within or adjacent to landslide-affected areas should be conducted to inform restoration strategies. This approach ensures that restored plant communities closely resemble their original state through near-natural restoration techniques.

In terms of plant community management, a staged configuration is recommended. In the short term, planting diverse native tree species (e.g., *Pinus armandii*, *Quercus robur*) and nitrogen-fixing shrubs (e.g., *Lespedeza bicolor*) can accelerate surface coverage. In the long term, thinning highly competitive species (e.g., *Juglans regia*) and supplementing shade-tolerant shrubs (e.g., *Euonymus alatus*) and herbaceous species (e.g., *Carex breviculmis*) can enhance understory diversity.

Regarding soil activity regulation, in the early post-landslide phase, adding organic matter or microbial inoculants should prioritize enhancing sucrase (SUC) and dehydrogenase (DHA) activities to address the lag in soil recovery. In the mid-term (after 6 years), monitoring soil potassium cycling and applying targeted fertilization can maintain the nutrient balance [57]. Through a synergistic model of “human-initiated intervention + natural succession”, the structural and functional recovery of post-landslide ecosystems can be significantly accelerated.

This study empirically demonstrates that near-natural restoration, when guided by time-staged management and functional trait monitoring, effectively promotes both biodiversity and ecosystem function recovery in landslide-affected forest systems. By integrating plant community traits, soil properties, and microbial activity across a restoration chronosequence, we show that early intervention with native species and targeted soil improvements can yield rapid ecological responses. Concurrently, long-term management strategies are crucial for sustaining structural complexity and functional resilience. A key implication of this work is the importance of balancing the timeliness of human intervention with the principles of natural succession [58]. Instead of a fixed intervention model, our findings advocate for a phased and adaptive management approach. Before restoration begins, comprehensive field assessments of dominant, fast-growing, and locally adapted species should be conducted in and around the landslide-affected areas. This identified species pool may then serve as the foundation for designing restoration schemes that closely mimic natural community composition and function.

## 5. Conclusions and Prospects

This study, through a comprehensive analysis of plant community species composition, α diversity, plant functional traits, and soil properties under near-natural restoration strategies in landslide-disturbed areas of Lantian County, innovatively reveals the key mechanisms and influencing factors across different time gradients in the post-landslide near-natural restoration process. The main conclusions are as follows: 1. Vegetation Recovery Dynamics: Near-natural restoration significantly accelerates vegetation reestablishment. After one year post-landslide, rapid colonization by native tree species resulted in species richness (D_m_) reaching 80% of that of the undisturbed control plots. Over extended restoration periods (6–11 years), competitive selection by dominant species led to a reduction in species number (e.g., tree layer species decreased from 10 to 3–5), but functional stability increased, with the canopy coverage in the 11-year plots reaching 95% of that of the control plots and the diameter at breast height increasing by 42%. 2. Nonlinear Soil Recovery: Soil nutrients and enzyme activities exhibited staged recovery. The first 1–2 years post-landslide represented a rapid response phase, with total nitrogen (TN) increasing by 132% and dehydrogenase activity (DHA) by 232%. From 6 to 11 years, recovery entered a stable accumulation phase, with available nitrogen (AN) reaching 79.62 mg/kg (approximately 85% of that of the control plots). 3. Plant–Soil Co-Evolution: In short-term restoration, the correlations between the vegetation and soil were weak. However, in long-term restoration (11 years), these correlations strengthened significantly (*p* < 0.05), with nitrogen and phosphorus cycling (AN, total phosphorus [TP]) and DHA enzyme activity showing strong associations with most plant traits, indicating the maturation of self-sustaining ecosystem mechanisms.

Near-natural restoration strategies provide an effective and sustainable approach for ecological recovery following landslides. Through appropriate substrate improvement, selection of native pioneer species, and optimization of plant functional configurations, near-natural restoration significantly enhances vegetation coverage, biodiversity, and soil environmental quality in landslide-disturbed areas. The current long-term observational studies and research on restoration effects across different site types remain limited. Future research should further explore restoration outcomes over longer time scales, as well as the impacts of varying site conditions and climatic factors on near-natural restoration effectiveness, and should optimize restoration techniques and management measures to achieve rapid, stable, and efficient ecosystem recovery post-landslide.

## Figures and Tables

**Table 1:** General information about experimental sites. (Wherein 1N denotes the 2021 landslide plot surveyed in 2022, 1NN denotes the 2021 landslide plot surveyed in 2023, and 1NCK and 1NNCK are their respective adjacent undisturbed control plots. 2N denotes the 2016 landslide plot surveyed in 2022, 2NN denotes the 2016 landslide plot surveyed in 2023, and 2NCK and 2NNCK are their respective adjacent undisturbed control plots. 3N denotes the 2011 landslide plot surveyed in 2022, 3NN denotes the 2011 landslide plot surveyed in 2023, and 3NCK and 3NNCK are their respective adjacent undisturbed control plots.)

Sample Group	Sample Areas	Slide Time (Year)	Slope Aspect	Gradient (°)	Altitude (m)	Coordinate	Sample Plot Details
1	1N (and NN)	2021	East	21~39	960	109.566717, 33.935510	Near-natural restoration
	1NCK (and 1NNCK)	/	South-east	16~38	938	109.555941, 33.923931	No landslides (CK)
2	2N (and 2NN)	2016	East	24~45	644	109.511463, 33.895653	Near-natural restoration
	2NCK (and 2NNCK)	/	South-east	16~34	620	109.517740, 33.887608	No landslides (CK)
3	3N (and 3NN)	2011	East	29~54	1084	109.498817, 33.906126	Near-natural restoration
	3NCK (and 3NNCK)	/	East	22~39	1065	109.498433, 33.905727	No landslides (CK)

**Table 2:** Summary of formulas.

Number	Title	Index	Formula	Parameter
1	Importance value	IV	$IV=\left(R_{c}+R_{h}+R_{f}\right)÷300$	$R_{c}$: relative coverage; $R_{h}$: relative height; $R_{f}$: relative frequency.
2	Margalef richness index	$D_{m}$	$D_{m}=\frac{M-1}{\ln N}$	M: number of community species; N: total individuals
3	Simpson advantage degree index	$D_{s}$	$D_{s}=1-\sum \frac{n_{i}\left(n_{i}-1\right)}{N\left(N-1\right)}$	$n_{i}$: number of individuals of species i; N: total individuals
4	Pielou evenness index	J	$J=\frac{H}{\ln M}$	M: number of community species; H: Shannon–Wiener diversity index
5	Shannon–Wiener diversity index	H	$H=-\overset{M}{\underset{i=1}{\sum }}P_{i}\ln P_{i}$	M: number of community species; $P_{i}$: Number of species i as a proportion of all species

**Table 3:** Species composition and importance value index of dominant species for different vegetation types in the 2021 landslide plots (1N and 1NN plots).

Vegetation Type	Sample Plot	Name	Importance Value IV%			Sample Plot	Name	Importance Value IV%		
			Tree IV%	Shrub IV%	Herb IV%			Tree IV%	Shrub IV%	Herb IV%
Tree	1N	*Pinus* *armandii*	22.42			1NN	*Toona* *sinensis*	23.74		
		*Zanthoxylum* *bungeanum*	21.14				*Zanthoxylum* *bungeanum*	23.01		
		*Toona* *sinensis*	15.07				*Pinus* *armandii*	19.96		
		*Quercus* *robur*	14.40				*Quercus* *robur*	12.29		
		*Salix alba*	11.27				*Salix alba*	8.72		
Shrub	1N	*Euonymus* *alatus*		50.34		1NN	*Lonicera* *caerulea*		55.77	
		*Kerria* *japonica*		21.76			*Cornus alba*		18.39	
		*Lonicera* *caerulea*		27.90			*Kerria* *japonica*		15.12	
							*Euonymus* *alatus*		10.72	
Herb	1N	*Artemisia* *annua*			30.54	1NN	*Artemisia* *annua*			23.20
		*Artemisia* *lavandulifolia*			23.41		*Artemisia* *lavandulifolia*			23.12
		*Duchesnea* *indica*			16.19		*Duchesnea* *indica*			13.80
		*Achnatherum* *chinense*			7.36		*Chrysanthemum* *lavandulifolium*			9.29
		*Phedimus* *aizoon*			6.89		*Phedimus* *aizoon*			8.68

**Table 4:** Species composition and importance value index of dominant species for different vegetation types in the 2016 landslide plots (2N and 2NN plots).

Vegetation Type	Sample Plot	Name	Importance Value IV%			Sample Plot	Name	Importance Value IV%		
			Tree IV%	Shrub IV%	Herb IV%			Tree IV%	Shrub IV%	Herb IV%
Tree	2N	*Pinus* *massoniana*	39.26			2NN	*Cotinus* *coggygria* var. *cinereus*	20.39		
		*Quercus* *variabilis*	24.89				*Pinus massoniana*	33.59		
		*Cotinus* *coggygria* var. *cinereus*	14.38				*Quercus variabilis*	23.77		
		*Fraxinus* *stylosa*	12.75				*Fraxinus stylosa*	13.07		
		*Quercus* *robur*	8.72				*Quercus robur*	9.18		
Shrub	2N	*Pueraria* *montana* var. *lobata*		62.98		2NN	*Pueraria* *montana* var. *lobata*		54.97	
		*Wikstroemia* *pilosa*		23.11			*Wikstroemia* *pilosa*		19.62	
		*Akebia* *trifoliata*		13.91			*Akebia trifoliata*		12.27	
							*Lespedeza bicolor*		7.74	
							*Berberis feddeana*		5.40	
Herb	2N	*Elsholtzia* *ciliata*			14.57	2NN	*Elsholtzia ciliata*			12.10
		*Humulus* *scandens*			11.63		*Humulus* *scandens*			10.39
		*Lagopsis* *supina*			5.67		*Lagopsis supina*			6.46
		*Artemisia* *argyi*			5.58		*Artemisia argyi*			5.77
		*Stellaria* *vestita*			5.43		*Artemisia* *lavandulifolia*			4.89

**Table 5:** Species composition and importance value index of dominant species for different vegetation types in the 2011 landslide plots (3N and 3NN plots).

Vegetation Type	Sample Plot	Name	Importance Value IV%			Sample Plot	Name	Importance Value IV%		
			Tree IV%	Shrub IV%	Herb IV%			Tree IV%	Shrub IV%	Herb IV%
Tree	3N	*Pinus* *bungeana*	32.23			3NN	*Gleditsia* *sinensis*	35.26		
		*Gleditsia sinensis*	31.17				*Pinus bungeana*	28.76		
		*Robinia pseudoacacia*	16.52				*Robinia* *pseudoacacia*	14.26		
		*Juglans regia*	13.42				*Juglans regia*	10.91		
		*Prunus* *davidiana*	6.66				*Prunus* *davidiana*	10.80		
Shrub	3N	*Spiraea* × *vanhouttei*		53.82		3NN	*Spiraea* × *vanhouttei*		57.95	
		*Euonymus* *alatus*		29.74			*Euonymus* *alatus*		25.88	
		*Lespedeza* *bicolor*		16.44			*Lespedeza* *bicolor*		16.17	
Herb	3N	*Carex* *breviculmis*			16.87	3NN	*Carex* *breviculmis*			14.20
		*Chrysanthemum* *lavandulifolium*			13.11		*Chrysanthemum lavandulifolium*			12.13
		*Artemisia* *argyi*			9.54		*Artemisia argyi*			8.33
		*Phedimus* *aizoon*			7.56		*Phedimus* *aizoon*			7.90
		*Artemisia* *eriopoda*			7.50		*Artemisia* *eriopoda*			6.42

## Data Availability

The data are not publicly available due to privacy or ethical restrictions.
